# Non-coding RNAs and neuroinflammation: implications for neurological disorders

**DOI:** 10.3389/ebm.2024.10120

**Published:** 2024-02-28

**Authors:** Yvonne Chen, Julia Mateski, Linda Gerace, Jonathan Wheeler, Jan Burl, Bhavna Prakash, Cherie Svedin, Rebecca Amrick, Brian D. Adams

**Affiliations:** ^1^ Department of Biology, Brandeis University, Waltham, MA, United States; ^2^ Department of RNA Sciences, The Brain Institute of America, New Haven, CT, United States; ^3^ Department of Biological Sciences, Gustavus Adolphus College, St. Peter, MN, United States; ^4^ Department of English, Missouri State University, Springfield, MO, United States; ^5^ Department of Electrical and Computer Engineering Tech, New York Institute of Tech, Old Westbury, NY, United States; ^6^ Department of English, Southern New Hampshire University, Manchester, NH, United States; ^7^ Department of Medicine, Tufts Medical Center, Medford, MA, United States; ^8^ Department of Biology, Utah Tech University, St. George, UT, United States; ^9^ Department of English, Villanova University, Villanova, PA, United States

**Keywords:** miRNA, ncRNA, cancer, Alzheimer, epilepsy, Huntington, neuroinflammation, neurology

## Abstract

Neuroinflammation is considered a balanced inflammatory response important in the intrinsic repair process after injury or infection. Under chronic states of disease, injury, or infection, persistent neuroinflammation results in a heightened presence of cytokines, chemokines, and reactive oxygen species that result in tissue damage. In the CNS, the surrounding microglia normally contain macrophages and other innate immune cells that perform active immune surveillance. The resulting cytokines produced by these macrophages affect the growth, development, and responsiveness of the microglia present in both white and gray matter regions of the CNS. Controlling the levels of these cytokines ultimately improves neurocognitive function and results in the repair of lesions associated with neurologic disease. MicroRNAs (miRNAs) are master regulators of the genome and subsequently control the activity of inflammatory responses crucial in sustaining a robust and acute immunological response towards an acute infection while dampening pathways that result in heightened levels of cytokines and chemokines associated with chronic neuroinflammation. Numerous reports have directly implicated miRNAs in controlling the abundance and activity of interleukins, TGF-B, NF-kB, and toll-like receptor-signaling intrinsically linked with the development of neurological disorders such as Parkinson’s, ALS, epilepsy, Alzheimer’s, and neuromuscular degeneration. This review is focused on discussing the role miRNAs play in regulating or initiating these chronic neurological states, many of which maintain the level and/or activity of neuron-specific secondary messengers. Dysregulated miRNAs present in the microglia, astrocytes, oligodendrocytes, and epididymal cells, contribute to an overall glial-specific inflammatory niche that impacts the activity of neuronal conductivity, signaling action potentials, neurotransmitter robustness, neuron-neuron specific communication, and neuron-muscular connections. Understanding which miRNAs regulate microglial activation is a crucial step forward in developing non-coding RNA-based therapeutics to treat and potentially correct the behavioral and cognitive deficits typically found in patients suffering from chronic neuroinflammation.

## Impact statement

miRNAs regulate cell-signaling regulatory networks at the post-transcriptional level, resulting in controlled cellular development responsive to extracellular stimuli. miRNAs also control the expression of genetic outputs during neuronal development. This neuronal cellular process is tightly regulated and tuned by acute and/or chronic inflammatory cues that, when disrupted, result in the onset of various neurological disorders. This review is an attempt to highlight miRNAs that modulate the neuroinflammatory state within the central nervous system and to elucidate the underlying mechanisms that promote or abrogate excessive inflammatory signaling as these biomolecules may become new therapeutic targets in the treatment of chronic neurological disorders such as epilepsy, ALS, Parkinson’s, Alzheimer’s, and Huntington’s.

## Introduction

Non-coding RNAs (ncRNAs) have emerged as crucial regulators in a multitude of biological processes, challenging the long-held view that RNA primarily functions as static templates of information utilized for ribosomal-mediated protein synthesis [1]. Over the past two decades, significant research has begun to unravel the seemingly mysterious yet crucial functions of ncRNAs which range from controlling developmental timing [2], hormone regulation [3], chromosomal inactivation [4], and the maintenance of normal cellular circuitry that restrains the onset of chronic disease [5]. The continued efforts by numerous scientific investigators have resulted in the development of ncRNA-based therapeutics for the treatment of those with HCV, hypercholesterolaemia, DMD, macular degeneration, neuromyelitis, myasthenia, as well as those with metastatic cancers [6].

In the mid-to-late 1990s researchers uncovered the complex mechanisms of small antisense RNA and how these ncRNAs could initiate gene suppression through Watson-Crick base pairing, in what is now termed RNAi (RNA inactivation) [7–11]. Continued elucidation of these RNAi pathways in the early 2000s indicated that ncRNAs specifically regulate the transcriptional abundance of mRNA and prevent the translation of an mRNA transcript into protein [12–15]. At this time, additional classes of ncRNAs were also becoming further characterized, such as tRNA, rRNA, snoRNA, snRNA, scaRNA, miRNA, lncRNA, and circRNA [16]. Advanced high-throughput sequencing technology identified the elevated abundance of certain ncRNAs in mammalian cells, and bioinformatic analysis further indicated the prevalence of sequence conservation from mammalian species to a wide variety of both invertebrate and vertebrate species [17–22]. The inferred importance of this ncRNA sequence conservation meant that seemingly complex cellular pathways in the mammalian system could instead be studied in genetically well-defined species such as *D. melanogaster* and *C. elegans* [23–26]. The identified ncRNA genetic targets could then be elucidated in mammalian cell cultures and further exploited for therapeutic purposes. For instance, miR-34 controls the abundance of *TP53* in *C. elegans*, and subsequent efforts confirmed that while miR-34a regulates *TP53* in human cell lines, miR-34a is also a crucial regulator of a host of genes that coordinately regulate the DNA repair response pathway [27–31]. Studies have now indicated thousands of conserved ncRNAs in every known vertebrate system, and revealed that ncRNAs are crucial in regulating several biological processes, including cellular proliferation, DNA editing, DNA modification, and cellular differentiation [32–39]. Taken together, this is compelling evidence that ncRNA molecules play critical roles in the control and maintenance of normal cellular and physiological processes.

### ncRNA regulatory potential

While ncRNAs have been implicated in regulating gene function within the cytoplasm due to the interaction of ncRNAs with ER-bound ribosomal polysomes, ncRNAs can also regulate RNA processing and translation, gene transcription, and cellular metabolic activity, indicating ncRNAs are functionally present in organelles such as the nucleus, ER, and mitochondria [40–44]. In one such example, Kim et al. explored the role of ncRNAs in the mitochondria and found *lncND5* interacts with MRPP1 and controls mitochondrial gene expression [45]. Additionally, *RMRP* interacts with PNPASE and GRSF1, which regulates mitochondrial RNA processing, mtDNA replication, and ultimately mitochondrial-mediated oxidative phosphorylation capacity. These findings have significant consequences in relation to the development of therapies for metabolic disorders, such as cancer. For instance, investigators demonstrated that cytosolic lncRNAs and miRNAs are essential for controlling the expression of glucose transporters GLUT1-3 as well as glycolytic enzymes via HK2, which results in dysregulated glucose uptake in response to insulin and limits the conversion of glucose to glucose 6-phosphate thereby reducing the overall metabolic activity of mammalian cells [46, 47]. Xu et al. also found that lncMMPA alters the levels of ALDH1A3, affecting the rate of aerobic glycolysis in HCC, yet promotes M2 macrophage polarization [47]. Some of these metabolically linked-ncRNAs are also functionally active in the nucleus, and interact with DNA regulatory machinery to control cellular proliferation in response to glucose stimulation [48, 49]. In fact nuclear RNAi, and nuclear miRNA/lncRNA molecules have a crucial role in regulating the abundance of nascent mRNA transcripts post-transcription, and modulate co-transcriptional alternative splicing activity [50, 51]. Together, these findings indicate that in response to an environmental stimuli, ncRNAs function within every cellular compartment to regulate essential cellular processes associated with the transcription and translation of genetic information, chromatin remodeling [52], RNA processing, and DNA damage repair [53], which in turn, affects the maturation and function of mammalian cells.

Given the ever increasing complexity of RNA terminology, the RNA community recognized miRNAs, lncRNA, and circRNA as ncRNAs that function as endogenous genetic regulators of mRNA abundance as well as controlling the expression and activity of all other ncRNAs (i.e., tRNA, rRNA, snRNA). While a majority of this review will focus on miRNA biology as a leading ncRNA therapeutic approach, it should be appreciated that lncRNAs were one of the original ncRNAs identified to have the capacity to silence genetic information [54–57]. X-chromosome inactivation (XCI) mediated by *XIST* is an example of how a lncRNA can control an unbalanced dosage of genetic information within the developing female embryo [58]. Furthermore, since lncRNAs are found in low abundance it is thought lncRNAs mostly act “*in cis*” or near their respective sites of transcription and subsequently co-transcriptionally regulate the expression of neighboring genes. The ENCODE project has identified over 10,000 lncRNAs, many of which are differentially expressed between normal and cancerous cells [59]. Over the past decade, lncRNAs have been found to interact with chromatin, modulate gene expression, function as protein decoys, and govern cellular physiology [60]. The crucial role of lncRNA in eukaryotic cell biology and in normal cellular function indicate these ncRNAs may also be novel targets for therapeutic intervention. However, because the mechanisms of biogenesis and mRNA silencing by miRNAs are more well-established, this review primarily focuses on the utilization of miRNA in the identification and treatment of neurocognitive disorders that currently have little to no curative options.

### miRNA biogenesis and function

**FIGURE 1:**
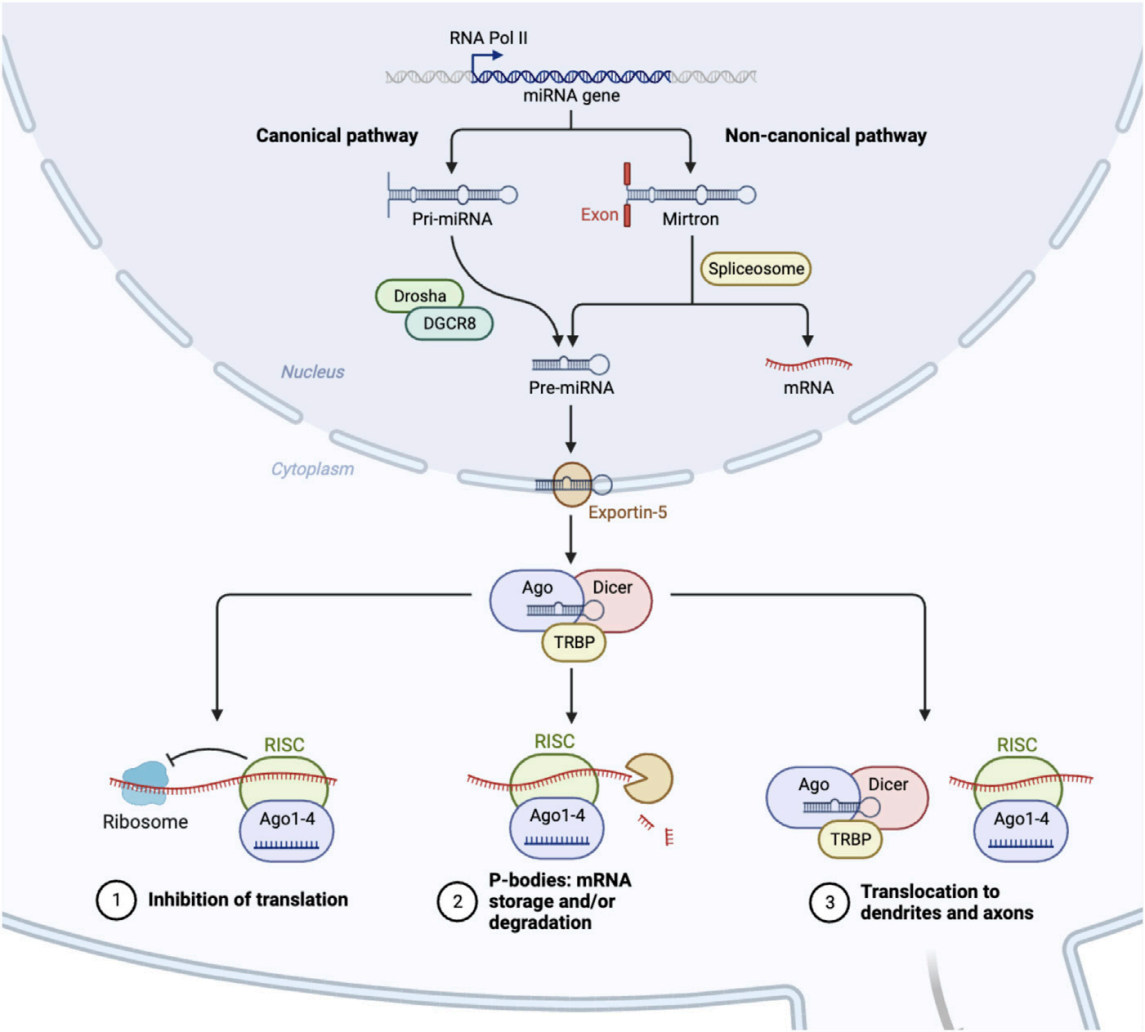
Biogenesis of microRNAs. In the nucleus, RNA polymerase II initiates the transcription of microRNAs (miRNA) [61]. As a result, this mono/poly-cistronic pri-miRNA serves as a substrate for enzymatic cleavage by Drosha/DCGR8, which is then exported from the nucleus as pre-miRNA [66]. A separate, non-canonical pathway involves the spliceosome, which cleaves intronic containing pre-miRNAs, and bypasses the Drosha/DCGR8 complex [67]. Pre-miRNA is further processed into a mature miRNA in the cytoplasm by the DICER enzyme [68]. This miRNA duplex subsequently binds with the protein AGO2 to form the RISC complex, which initiates mRNA degradation at specific locations of mRNA transcripts, stalls polysome machinery preventing translation, or transports miRNAs to various subcellular regions [68].

miRNAs are small ncRNAs that modulate gene expression by binding to complementary sequences predominantly within the 3′UTR of target mRNAs [61, 62]. Similar to siRNA technology, the mechanism of action(s) linked to miRNAs indicates that miRNA/RNAi-based therapies could be used to silence, and in some cases repair, any misregulated genetic element within the human genome to correct a specific pathological deficit. miRNAs are, however, distinct from siRNAs in that they are endogenously expressed in the genome and transcribed in the nucleus by RNA polymerase II/III [63–65] (see Figure 1). The resulting primary miRNA (pri-miRNA) transcript is unique in that the pri-miRNA folds creating hairpin bulges similar to tRNA, which is recognized by the RNaseIII enzyme DROSHA [69–71]. Pri-miRNAs can originate from large intergenic regions of the genome, (i.e., areas with little protein coding activity), as well as from intronic regions, (i.e., from sequences processed and spliced out of newly transcribed mature mRNAs), and sometimes are polycistronic and contain multiple mature miRNA sequences [72]. Recently, a non-canonical pathway was identified that further categorized a subset of intronic miRNAs known as “miRtrons” (see Figure 1). These miRNA sequences mapped precisely to short hairpin introns and spanned each of the exon-intron junction borders [67, 73], meaning that when the spliceosome recognizes these junctions, a miRNA will be concomitantly produced with the exonic mature mRNA. Ultimately these findings indicate that an intronic sequence is no longer considered “junk DNA” and in some cases an entire intron codes for a miRNA that has significant downstream regulatory potential.

Canonical pri-miRNAs, as well as miRtrons, both contain sequential hairpin bulges, a terminal loop, are ∼85 nucleotides in length, and recognizable by the microprocessor complex containing DROSHA and cofactor DGCR8 (see Figure 1). In *D. melanogaster*, DGCR8 is called Pasha, yet performs similar functions [74–76]. The Drosha-DGCR8 heterodimer recognizes approximately one to two helical RNA turns of sequence from the pri-miRNA stem and splits the phosphodiester bond, releasing a short hairpin-shaped RNA ∼60 nucleotides in length [66]. These canonical pre-miRNAs and the pre-miRNAs generated from the miRtron pathway are exported to the cytoplasm via Exportin-5, and undergo further processing by the RNAse-III enzyme, Dicer [68, 77, 78]. Dicer cleaves the double-stranded pre-miRNA approximately two helical turns from the terminal loop [79], generating short double-stranded RNA fragments ∼22 nt in length known as the mature miRNA duplex. This duplex contains a guide and a star strand offset on the 5′ ends, allowing for strand selection to occur via Argonaute and the RNA-induced silencing complex (RISC) [80–82].

The focus on the miRNA biogenesis pathway is an important area of research, as many second and third generation siRNA therapeutics utilize the miRNA biogenesis pathway to induce a particular outcome of gene repression, activation, or editing [83–85]. Duplexed siRNAs will also undergo strand selection, and can be engineered with sequence bias to favor a particular guide siRNA. Both the guide miRNA or synthetic siRNA is incorporated into RISC which contains, AGO/TRBP and binds to complementary mRNAs within the 3′ UTR resulting in stalling of polysomal translational machinery, the degradation of the mRNA through slicing activity of AGO2, the storage or degradation of mRNA in P-bodies, or the transport of mRNA to exosomes or other cellular regions, such as dendrites (see Figure 1) [86–89].

While much attention has been given to the role of miRNAs in posttranscriptional gene silencing, recent studies have highlighted the broader functions of miRNAs in regulating nuclear chromatin dynamics. Catalanotto et al. highlight the nuclear function(s) of miRNAs, including their involvement in transcriptional regulation and their ability to recognize gene targets at the promoter level [90]. Politz et al. investigated the presence and localization of miRNAs within the nucleolus, and observed that certain nucleolar miRNAs contribute to ribosome biosynthesis and associate with the granular component, where ribosome assembly occurs [91]. Interestingly, in Alzheimer’s patients, reduction in nucleolar size is notable, concomitant with reduced rRNA transcription [92]. In Parkinson’s, nucleolar integrity is maintained by ɑ-synuclein co-localization with nucleolin, which is regulated by the presence of miR-7 and miRNA-153 [93, 94]. Therefore, miRNAs may regulate the pathophysiology of many neurological disorders, absent of the canonical gene repression mechanisms mentioned above. In fact a number of synthetic RNAi-based therapies have utilized this approach to generate oligonucleotide based therapies such as AMT-130, and VY-HTT01 to correct the HTT gene defect in individuals with Huntington’s [95], as well as antisense mediated regulation of ATXN and CAG repeats in individuals with spinocerebellar ataxia [96].

### miRNAs and neuroinflammation

Neuroinflammation is a complex homeostatic process that is essential in mitigating exogenous pathogens from entering the central nervous system (CNS) [97]. Normally, pro-inflammatory responses allow for the acute recruitment of macrophages, natural killer cells, and T-cells to attack these pathogens in a robust manner [98]. Once the pathogen is removed, inflammatory cytokine signaling decreases, and the presence of inflammatory cells diminishes. A key factor in the inflammatory process is the activation of microglia, which safeguards the brain from neuronal damage through the release of neuroprotective cytokines and other inflammatory factors [99–101]. Chronic neuroinflammation is the continued presence of inflammatory cytokine signaling absent of a pathogen, which results in a persistent stress response within the microglia of the CNS, causing disruptions in normal neuronal communication, damage to the neuronal circuitry, and neurodegeneration [102]. The etiology of various neurodegenerative diseases such as Alzheimer’s, Parkinson’s, amyotrophic lateral sclerosis (ALS), and certain forms of epilepsy, are all thought to involve a neuro-inflammatory component.

Recently, the heterogeneic classification of the microglia into M0, M1, M2a, M2b, and M2c cell types indicate that specific microglial cells are responsible for initiating differential cellular responses during neuronal perturbations [103]. For instance, M2 cells tend to secrete anti-inflammatory cytokines and neurotrophic factors, as well as support oligodendrogenesis and remyelination [104]. By contrast, M1 cells respond to bacterial byproducts such as LPS and IFN-y, causing a release of TNF-α and IL-6 inducing oxidative stress and neuronal dysfunction [105]. Given the genetic basis for classifying microglial cell types has been established, researchers can now elucidate how M1 cells remain activated post infection, or how M2 cells are prevented from secreting anti-inflammatory cytokines that promote neurogenesis. One of these regulatory processes involves ncRNA, since miRNA activity and expression are repeatedly involved in cell-fate decisions during cellular differentiation [106–108]. Elucidating the mechanisms of M0 to M1 or M2 cell type transition would be an important area of study so as to further develop candidate therapeutics to delay or abate the progression of neurodegenerative disease.

**FIGURE 2:**
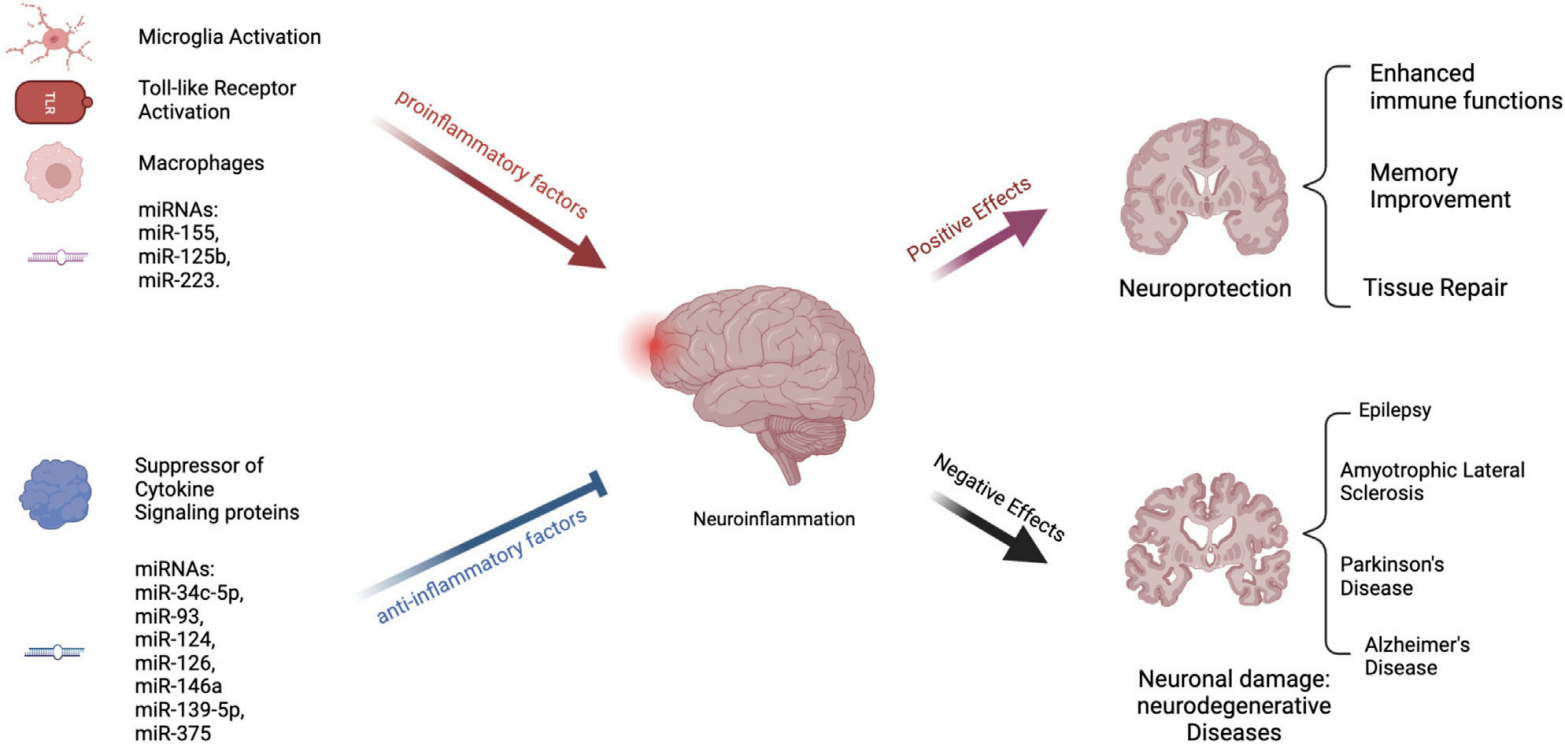
Process of Neuroinflammation. Several pathways exist that can either stimulate or decrease neuroinflammation. Inflammation is encouraged by proinflammatory substances. These include the toll-like receptors, macrophages, and microglial activation [108]. Both animal and *in vitro* studies have suggested that some microRNAs, such as miR-155, miR-125b, and miR-223, are proinflammatory [101, 110, 111]. On the other hand, suppressors of cytokine signaling proteins are anti-inflammatory [110]. Inflammation is also reduced by many miRNAs, including miR-34c-5p, miR-93, miR-124, and others [112–116]. Chronic neuroinflammation has been linked to the development of neurodegenerative illnesses such as epilepsy, Amyotrophic Lateral Sclerosis (ASL), Parkinson’s Disease (PD), and Alzheimer’s Disease (AD) [108, 117–121].

Independent of the bifurcation of microglial cell-fate, miRNAs also play a significant role in the activation or suppression of neuroinflammation by controlling the abundance of central mediators of NLRP3 priming and/or activation of the inflammasome [109] (see Figure 2). For instance, miR-155 regulates the activity of NF-kB, a major protein complex that controls DNA transcription and cytokine production, supports maturation of dendritic cells, promotes recruitment of neutrophils, stimulates M1 polarization of macrophage and microglial cells, and activates T- and B-cells [122]. Interestingly, the miR-155 host gene is found within the *BIC* gene cluster, a region that contains transcription factor binding sites for NF-kB, SMAD4, and other interferon regulatory factors [123, 124]. As expected, miR-155 is expressed in a coordinated feedback loop with a series of transcription factors that contain miRNA bindings sites for miR-155 [125]. This regulatory loop ensures robust gene expression of pro-inflammatory cytokines is rapidly expressed during an acute infection, while simultaneously activating a regulatory fail safe to dampen unabated inflammatory signaling. Guil et al. identified a similar feedback mechanism in which RNA-binding protein hnRNP A1 modulates the processing of miR-18a [126]. Specifically, hnRNP A1 binds exclusively to pri-miR-18a, facilitating DROSHA processing, in turn promoting a rapid depletion of hnRNP A1 which results in diminished miR-18a processing and reduced pre-miR-18a levels.

Since these initial discoveries, hundreds of miRNAs have been identified to either stimulate or abrogate anti-inflammatory responses (see Figure 2) [127]. For instance, miR-223, is highly expressed in the hematopoietic system and regulates granulopoiesis, a process essential in differentiating bone-marrow derived myeloblasts so as to re-generate the depleted neutrophil reservoir during and after an acute infection [128]. However, miR-223 keeps neutrophils and monocytes in an inactive state, absent of inflammatory stimuli. Another anti-inflammatory miRNA, miR-124, controls the levels of TNF-a in the microglia, dampens the abundance of reactive oxygen species, and promotes M2 type polarization [129, 130]. Identifying these anti-inflammatory miRNAs is the first step in the process of developing oligonucleotide based therapeutics to maintain the activity of healthy microglia and promote neuronal differentiation. In support of this approach, imbalances in miRNA levels, such as miR-155, miR-18a, and miR-124 results in heightened inflammation *in vivo*. In zebrafish, levels of miR-18a spike one to 5 days post retinal injury [131]. In miR-18a^mi502^ altered animals, photoreceptor regeneration was delayed, and was fully restored once the continued presence of active inflammation was abated by dexamethasone treatment. In mice, the loss of miR-223 results in a protective phenotype using a MOG induced EAE model [101, 132] These mice exhibit decreased spinal cord inflammation and reduced demyelination. This seems counterintuitive, yet using a stable miR-223 knockout model, developmentally, these mice most likely have a depleted neutrophil reservoir. Reducing the concentration of miR-223 also results in the decline of inflammatory responses and the presence of increased autophagy within the microglia. miR-155 has been extensively studied using a variety of knockout and overexpression models. In mice it is clear that miR-155 modulates the polarization of the microglia and can promote neuroinflammation by inhibiting inflammasome signaling mediators such as SIRT1 and SOCS1, and by promoting IL-6 production [110, 133]. Specifically, in APPtg and 3xTg Alzheimer mouse models, the persistently elevated levels of miR-155 was associated with the proliferation of dendritic cells, as well as T regulatory cells that support neuroinflammation.

While proper miRNA production leads to healthy cell function, regulation of gene expression, and maintenance of normal intracellular pathways, the disruption of miRNA biogenesis can also contribute to a variety of illnesses (see Figure 2). Tang et al., identified miR-709 as a nuclear miRNA involved in the regulation of miR-15/16 biogenesis [134]. Specifically, miR-709 binds to pri-miR-15a/16-1, ∼0.8 kB away from the pre-miRNA hairpin bulge, and recruits chromatin remodeling complexes that prevents the processing of pri-miR-15a/16-1 by DROSHSA. Interestingly, both miR-15/16 and miR-709 have important roles in controlling neuroinflammation *in vivo*. miR-709 controls glutamatergic signaling and endosomal trafficking in cortical neurons, resulting in balanced sleep-wake cycles [135]. The deletion of mir-15/16 in T-regulatory cells results in animals with extensive lymphoproliferative disease and systemic tissue inflammation [136]. Some of the phenotypes could be linked to the abundance and activity of Toll-like receptors (TLRs), which are expressed in dendritic cells, and macrophages, as well as epithelial cells. TLRs are the first line of defense against pathogens by recognizing pathogen-associated molecular patterns (PAMPs) [137]. When activated, TLRs trigger signaling pathways that involve various molecules and transcription factors, leading to the release of inflammatory cytokines. In the central nervous system, TLRs are present mainly in microglia and astroglial cells, but also in some neurons and other glial cells. While TLR activation is a defense mechanism against invaders and tissue damage, excessive activation can disrupt the balance of immune responses, resulting in the production of persistent pro-inflammatory molecules [138]. This can lead to neuroinflammation and damage in the nervous system, contributing to various neurodegenerative diseases. miR-15a, has been shown to target TLR4-associated pathways in bone marrow derived macrophages affecting the mortality of mice using a model of sepsis [139] (see Figure 2). Furthermore, miR-155 activity is induced by TLR signaling, while miR-146a was reported to control cytokine and TLR signaling through a negative feedback regulatory loop with IRAK1 and TRAF6 [140, 141]. Taken together, these studies indicate, miRNAs can intersect with TLR signaling and modulate the process of an inflammatory response. Specifically, when TLR4 is activated by substances that are harmful to the nervous system, such as LPS, a pattern of expression that includes numerous miRNA clusters, such as miR-15, miR-182, and miR-200, result in a coordinated effort to stimulate acute inflammatory response to remove the pathogen. However, dysregulated miRNA signaling can result in persistent inflammatory cues that promote a diseased physiological state by promoting neuroinflammation. Here we discuss relevant neurological disorders that are the result of mis-regulated miRNA expression and activity, which could serve as the basis for therapeutic intervention using oligonucleotide therapeutics to abate or slow the onset of these diseases.

### miRNAs and epilepsy

Epilepsy is a group of disorders characterized by recurrent seizures [142], and is one of the most common neurological conditions that affects at least 50 million people worldwide [143, 144]. Epilepsy follows a bimodal age distribution pattern, meaning that infants and elderly are at the highest risk, with the highest incidence rate being in individuals over 70 years of age [145]. Onset rates in young children have decreased with an increase in perinatal care and sanitation, leading to a decrease in infectious disease. However, epilepsy rates in the elderly have increased due to increased life expectancy, resulting in higher rates of other neurological disorders and cancers that may contribute to the etiology of epilepsy [146]. The International League Against Epilepsy (ILAE) defines the disorder as a patient having 1) two seizures, separated by more than 24 h, occurring without a clear cause, 2) one seizure without a clear cause, and the chance of having more seizures in the next 10 years being over 60%, or 3) diagnosis of an epilepsy syndrome [147]. Although the definition of epilepsy suggests that it is a single disorder, it is more accurate to describe epilepsy as a group of disorders with diverse etiologies and outcomes. Different epilepsy syndromes are characterized by seizure type(s), age of onset, developmental status, comorbid features, and etiology [142]. The ILAE has defined six etiologic categories; Structural, Genetic, Infectious, Metabolic, Immune, and Unknown [148]. Understating the exact genetic underpinnings that manifest these specific etiologies is crucial in understanding how to better treat individuals with epileptic syndromes.

#### Genetic etiology

The genetic etiology of epilepsy is still controversial [149]. The underlying pathogenesis involved in epilepsy include ion channel dysfunction, synaptic remodeling, gliosis, and neuronal death [117, 144]. Genetics influence ∼70% of epilepsy cases, while acquired and environmental factors, such as traumatic injury and infections of the brain account for the remainder of cases. Genetic evidence for epilepsy onset arises from familial studies that have identified rare genetic variants in genes coding for solute carrier channels, ligand-gated channels, GABAergic receptors, and DNA repair genes [142, 150, 151]. For instance, mutations in GABRA1 at 5q34-q35, as well as single nucleotide polymorphisms in BRD2 are more prevalent in individuals with juvenile myoclonic epilepsy when compared to healthy individuals. Another example involves DEPDC5-related epilepsy, in which frameshift mutations in DEPDC5 result in improper complex formation with the GATOR1 complex, a negative regulator of mTORC1. The resulting dysregulation in mTORC1 signaling results in the phenotypes associated with family-related focal epilepsy [152].

As these genetic players are further identified through GWAS and genetic linkage studies, the role of miRNA as pleiotropic regulators of epilepsy becomes increasingly obvious. Firstly, there is evidence that epilepsy is a heterogeneous entity, meaning that multiple alleles may be causing the disease rather than a single variant [142]. Secondly, DEPDC5, mTORC1, and GABRA1 are all direct targets of various miRNAs (see Table 1), and the resultant aberrant expression of these genes due to post translational regulation by miRNAs may explain why mutational linkage studies have under reported the prevalence of certain epileptic subtypes. Further evidence supporting this notion is the finding that miR-106b, miR-146a, and miR-301a are highly detectable in the serum from epileptic patients, and miR-181a regulation of GABRA1 results in attenuated viscero-motor response in the spinal cord of rats [158]. Further mechanistic study is required to elucidate the contribution of each miRNA to the genetic candidates implicated by genetic linkage mapping. This will be a painstaking process, yet will result in the development of valid miRNA-based epileptic therapies.

**TABLE 1:** List of miRNAs and their implications involved in neurological disease.

Disease	miRNA	Target	Implications
Epilepsy	miR-146a	TLR4-NFκB pathway	Up-regulated in epilepsy; Acts as a negative feedback regulator; Dampens NFκB activity; Reduces IL-1 and IFN-α production; Mitigates inflammatory responses post-seizure
	miR-155	Anti-inflammatory regulators, TNF-α	Pro-inflammatory; Induced through NFκB after TLR stimulation; Enhances neuroinflammation by inhibiting anti-inflammatory regulators; Up-regulated in chronic TLE
	miR-125a-5p	CAMK4	Down-regulated in PTZ-induced epilepsy rats; Overexpression reduces seizures and inflammatory factor levels; Alleviates epileptic seizure and inflammation
	miR-34c-5p	HMGB1	Down-regulated in drug-resistant epilepsy (DRE); Exacerbates neuroinflammation; Aggravates hippocampal neuron loss
	miR-27a-3p	Apoptosis regulators, IL-1B, IL-6, TNF-α	Up-regulated in epileptic mice; Inhibitor relieves seizures and prevents apoptosis of hippocampal neurons; Inhibits inflammatory response; Reduces serum levels of IL-1β, IL-6, and TNF-α
	miR-139-5p	MRP1	Down-regulated in refractory epilepsy; Targets MRP1; Implicated in multidrug resistance
	miR-153	HIF-1α	Dysregulation implicated in refractory epilepsy; Possibly regulates HIF-1α expression
	miR-15a-5p	Unknown	Down-regulated in children with TLE
	miR-21-5p	STAT3, PDCD4, IL-6	Down-regulated in epilepsy; Up-regulated STAT3 in epileptic rats; Suppresses expression of STAT3, decreases IL-6 levels, reduces hippocampal neuron loss; Inhibits apoptosis by regulating PDCD4
	miR-134	MAP3K9	Promotes apoptosis; Reduces MAP3K9 levels
ALS	miR-34a	RNA metabolism, Aggregation	Dysregulated in ALS; Alters RNA metabolism; Implicated in protein aggregation
	miR-504	RNA metabolism, Aggregation	Dysregulated in ALS; Alters RNA metabolism; Implicated in protein aggregation
	miR-124	Motor neuron degeneration	Implicated in ALS progression; Associated with motor neuron degeneration
	miR-155	Inflammatory impact	Implicated in ALS progression; Has inflammatory impact
	miR-206	ALS pathology	Dysregulated in ALS pathology; Implicated in ALS-relevant pathways
	miR-27a	ALS pathology	Dysregulated in ALS pathology; Implicated in ALS-relevant pathways
	miR-338-3p	Biomarker potential	Potential ALS biomarker; Circulating miRNA
	miR-183	Biomarker potential	Potential ALS biomarker; Circulating miRNA
	miR-451	Biomarker potential	Potential ALS biomarker; Circulating miRNA
	miR-125a/b	Biomarker potential	Potential ALS biomarkers; Circulating miRNAs
	TDP-43 binding miRNAs	Dysregulation	Dysregulated in ALS patients; Indicates TDP-43 dysfunction
Parkinsons	miR-124	MEKK3, p62, p-p38	Regulation of neuroinflammatory response in microglia, Decreased expression in PD models, Overexpression reduces pro-inflammatory cytokines, Increases secretion of neuroprotective factors
	miR-155	Unknown	Increased expression in mouse models of PD, Plays a key role in the inflammatory response to α-syn, Neurodegeneration related to inflammation in PD
	miR-375	SP1	Protects dopaminergic neurons, Reduces neuroinflammation and oxidative stress in PD
	miR-7	α-synuclein	Protects cortical neurons from MPP+ toxicity, Interferes with MPP+ induced down-regulation of mTOR
	miR-153	α-synuclein	Protects cortical neurons from MPP+ toxicity, Interferes with MPP+ induced down-regulation of mTOR
	miR-34b/c	α-synuclein	Represses α-synuclein expression, Decreased levels in PD patient brains, Misregulated in pre-clinical PD brains, Down-regulation leads to mitochondrial dysfunction, Increases oxidative stress, contributing to PD
Alzheimers	miR-9	Unknown	Elevated levels in AD brains. Potential role as a biomarker for AD.
	miR-34a	TREM2	Increased levels in AD. Impairs microglial phagocytosis and increases cytokines. Potential therapeutic target for restoring TREM2
	miR-125b	Bcl-W, DUSP6, PPP1CA	Elevated in AD cerebrospinal fluid (CSF). May increase neuroinflammation and tau hyperphosphorylation
	miR-146a	Unknown	Elevated levels in AD brains
	miR-155	Unknown	Elevated levels in AD brains. Plays a crucial role in regulating memory loss
	miR-30b	Unknown	Highly upregulated in AD brains. Dysregulation may lead to cognitive and synaptic decline
	miR-124	PTPN1	Elevated levels in AD hippocampus. Dysregulation affects brain dysfunction in AD.
	miR-132	C1q	Reduced in AD brains. Dysregulation increases AD deposition
	miR-155	IL-1ß, IL-6, TNF-α	Elevated levels in AD hippocampus. Inhibition may decrease apoptotic protein Caspase-3
Huntington’s Disease	miR-137	Unknown	Downregulation is pathological in HD, ameliorates mHtt-induced phenotypes [153]
	miR-1010	Unknown	Downregulation is pathological in HD, ameliorates mHtt-induced phenotypes [153]
	miR-219	Unknown	Detrimental in HD model and control flies, unrelated to HD pathology [153]
	miR-10	Unknown	Exacerbates phenotypes in HD flies and affects eclosion rate in controls [153]
	miR-305	Unknown	Alleviates phenotypes in HD flies, detrimental effects in control flies [153]
	miR-132	Unknown	Enhances motor function and lengthens lifespan in HD mice [154]
	miR-34a-5p	NDUFA9, TAF4B, NRF1, POLR2J2, DNALI1, HIP1, TGM2, POLR2G	Direct target genes, potential for future therapeutic interventions [155]
	miR-10b-5p, miR-10b-3p, miR-302a-3p	Unknown	Associated with neuropathological involvement and striatal scores [156]
	miR-10b-5p	Unknown	Strongly over-expressed in HD cases, related to CAG length-adjusted age of onset [156]
	miR-9*	Unknown	Lower expression in HD patients, involvement in HD pathogenesis [157]

#### Immune etiology

Neuroinflammation is another significant contributor to the pathogenesis of epilepsy, particularly temporal lobe epilepsy, which is the most common cause of partial seizures and the primary source of refractory seizures [117–119]. Stimulation of pro-inflammatory molecules such as IL-1*β*, IL-6, TNF-ɑ, and TLRs induces neurological modifications that result in impaired integrity of the blood brain barrier, neuronal loss, and hyper-excitotoxicity. Such pathological events transform the normal brain into an epileptic state, with an imbalanced functioning of excitatory and inhibitory neurons (see Figure 2). Systemic presence of proinflammatory cytokines result in limited hippocampal neurogenesis, upregulation of AMPA receptors in glial cells resulting in neurotoxicity, active MMP9 that causes narrowing of dendritic protrusions reducing synaptic plasticity, and VEGF-mediated leakage of monocytes into the brain parenchyma [119]. The continued identification of new mechanisms of action in the epileptic brain offers new avenues for developing novel antiepileptic therapies.

Besides electroconvulsive therapy, most of the original antiepileptic therapies were really antiseizure medications (i.e., valium) designed for muscle spasms with contraindications for effective use in epileptic patients [159]. Although these drugs suppress seizure activity, they have no effect on the underlying pathophysiology in restoring imbalanced functioning of excitatory and inhibitory neurons. Third generation anticonvulsants such as brivaracetam, levetiracetam, and perampanel, developed after 2010 had the efficacy to regulate glutamate receptor excitation, AMPA activity, and/or synaptic vesicle glycoprotein 2A uptake. While these seemingly more effective anti-inflammatory agents were developed to antagonize the manifestation of the epileptic brain, (i.e., the epileptic seizures), these treatments do little to hinder or reverse the process of epileptogenesis. Given miRNAs are crucial pleiotropic suppressors of aberrant inflammatory activity, as we have discussed earlier in this review, the potential for miRNAs to be used as the next-generation of anti-epileptic therapies looks promising. Furthermore, the utilization of miRNAs might resolve the problem associated with refractory epileptic episodes [160].

#### Mechanistic basis for miRNA-based epilepsy therapies

miRNAs are involved in the onset, development, and progression of epilepsy by regulating many of the key pathological processes mentioned above [118, 119]. For instance, miR-134 control of dendritic spine morphology [161], and the depletion of miR-134 after an epileptic seizure results in reduced neuronal spines and an induced neuroprotective environment. Furthermore, miR-134 overexpression promotes apoptosis by reducing MAP3K9 mRNA and protein levels after an epileptic seizure, which affects the occurrence and latency of future seizure events. In patients with terminal lobe epilepsy, miR-134a serum expression tends to be lower than those without epilepsy, yet these levels increase from those suffering drug-resistant epilepsy. This could be explained by the finding that miR-134a causes reductions in CA3 pyramidal neurons potentially via the gene target CREB.

Another important anti-inflammatory miRNA and one of the most widely studied miRNAs in epilepsy, miR-146a, is abnormally expressed in both affected patients and in animal models of the disease (see Table 1). miR-146a is notably up-regulated in the serum of individuals with focal and generalized epilepsy, as well as within a pilocarpine-induced epileptic model [150]. miR-146a is a significant negative feedback regulator of the TLR4-NFκB proinflammatory pathway. Following a seizure, activated TLR4 triggers proinflammatory NFκB signaling and up-regulation of IL-1, IFN-α, and miR-146a. The increase in miR146a levels dampens NFκB activity, reducing IL-1 and IFN-α production, resulting in mitigated inflammatory responses post-seizure [117, 150]. Tao et al. used a lithium-pilocarpine mouse model to evaluate whether intranasal delivery of miR-146a mimics could delay seizure onset of terminal lobe epilepsy [118]. Using this therapeutic approach, miR-146a overexpression resulted in an increased percentage of animals having no induced seizure activity, extended latency of generalized convulsions, and reduced hippocampal damage. Inflammatory modulators such as, NF-*κ*B, TNF-*α*, IL-1*β*, and IL-6 were also reduced, supporting an anti-inflammatory role of miR-146a via modulation of the TLR4-NFκB pathway [118].

While epilepsy is mostly described as a neurotransmitter imbalance, the consequence of chronic seizure activity is neuronal apoptosis, reorganization of neuronal synapses, and formation of abnormal synaptic loops that promote epilepsy recurrence. miRNAs play a crucial role in regulating both of these pro-apoptotic and pro-inflammatory pathways. As an example, miR-27a-3p expression is elevated in the hippocampus of epileptic mice [162]. Lu et al. used *in vivo* and *in vitro* models of epilepsy induced with kainic acid to determine the effect and mechanism(s) of action for miR-27a-3p [162]. Specifically, hippocampal tissue samples from rats treated with kainic acid had elevated miR-27a-3p expression, while the presence of a miR-27a-3p inhibitor during this treatment relieved kainic acid induced epileptic seizures. Additionally, the use of a miR-27a-3p inhibitor prevented apoptosis of cultured hippocampal neurons from epileptic rats. ELISA testing further revealed that, compared with the control group, proinflammatory factors IL-1B, IL-6, and TNF-α were significantly increased after intraperitoneal kainic acid treatment. The utilization of miR-27a-3p inhibitor co-treatment during this procedure diminished the activity of kainic acid induced pro-inflammatory response, as evidenced by reduced serum levels of IL-1β, IL-6, and TNF-α [162]. Taken together, the development of miRNA inhibitor based treatments or oligonucleotide therapies directed at the gene targets of miRNAs, such as miR-27a, may result in sustainable decreases in mortality and morbidity associated with chronic epileptic seizures.

#### miRNA and drug resistant epilepsy

In a 2022 review, Ghafouri-Fard et al. reported that several miRNAs were expressed and functionally active in refractory epilepsy, as defined by a lack of response to anti-seizure medication(s). Firstly, miR-139-5p expression is reduced in children with refractory epilepsy, concomitant with the up-regulation of multidrug resistance-associated protein 1 (MRP1) [113]. Similar expression patterns have been identified in brain tissue samples from rat models of refractory epileptogenesis, and *in vitro* studies have confirmed MRP1 is targeted and repressed by miR-139-5p. Moreover, rats transfected with miR-139-5p plasmids had enhanced neuronal survival, blunted neuronal injury, and re-sensitization to phenobarbitone treatment when compared to rats that underwent amygdala kindling with continual phenobarbitone exposure alone.

Fu et al. also investigated the mechanisms underlying the neuroinflammatory process in refractory epilepsy [112]. They found miR-34c-5p was significantly down-regulated in patients with drug-resistant epilepsy compared to controls. Experiments in kainic acid-induced epileptic rat models have shown that the subsequent down-regulation of miR-34c-5p and up-regulation of the inflammation-related mediator genes HMGB1 and IL-1β, post treatment, contributed to neuroinflammation and hippocampal neuron loss [112, 113]. Luciferase reporter assays *in vitro* confirmed that HMGB1 and hypoxia-inducible factor-1α (HIF-1α) were just some of the genetic targets of miR-34c-5p [112]. The continued efforts of numerous investigators into the mechanisms of miRNA function in epileptogenesis have culminated into the development of bioinformatic tools such as EpimiRBase [160], that depicts theoretical as well as experimentally verified, miRNA-gene target interactions that modulate the cellular mechanisms contributing to epileptogenesis (see Table 1). Many of these miRNA targets are also regulators of neuroinflammation, apoptosis, and angiogenesis.

Taken together, these findings indicate that while miRNAs such as miR-139-5p and miR-34 are being developed independently as therapeutics for certain types of metastatic cancer, these miRNAs should also be considered as candidate biomolecules for the next-generation of antiseizure medications for those suffering from recurrent epilepsy.

#### The development of genetically modified miRNA animal models of epilepsy

As mentioned earlier, a number of animal models have been developed to assess the underlying etiology of epilepsy. For instance, inhalation of flurothyl and electroshock treatment in rats are models that mimic the seizure phenotypes associated with epilepsy. Unfortunately these models do not identify the genetic onset of the disorder. Intraperitoneal injection of PTZ, the use of NMDA, phenobarbitone injections, or the intracortical injection of iron are more stable model systems that can be employed to investigate genetic variants that contribute to epileptogenesis. This is an important concern, because the stochastic regulatory potential of miRNAs can not be properly studied using unstable models of any genetic disorder, including epilepsy. Fortunately, using a lithium-pilocarpine rat model that mimics mesial temporal lobe epilepsy (MTLE), Ashhab et al., found an intricate connection between TNF-α expression and miR-155 levels [163]. In children with chronic TLE, the expression levels of both miR-155 and the pro-inflammatory cytokine TNF-α are increased [117]. Furthermore, in cultured hippocampal astrocytes, LPS treatment or activation of MRP8 resulted in elevated levels of both miR-155 and TNF-α. Conversely, lenalidomide treatment, a compound known to activate NK cell-mediated cytotoxicity, resulted in the suppression of miR-155 and TNF-α. Further investigation revealed that TNF-α functions as a positive feedback signal that transcriptionally regulates miR-155 expression.

Despite the unstable animal models of epilepsy, it is not surprising that miR-155 was found to be differentially expressed during the varied inflammatory states of epileptogenesis. miR-155 is a *bona fide* pro-inflammatory miRNA and regulates almost every aspect of immune cell functions by controlling the levels of SMAD2, IL-13α, LXRa, and SHIP-1, allowing for the unabated production of TNF-α, IL-6, IL-8, and the polarization of macrophages [163]. miR-155 also controls dendritic cell maturation, promotes differentiation and development of Th17, Th1, and T-regulatory cells, as well as stimulates the proliferation of mature B-cells and germinal center formation. Within microglia and resident macrophages, miR-155 also activates NFκB and TLR signaling resulting in the secretion of pro-inflammatory cytokines such as IFN-γ. Together, these findings support the notion that chronic neuroinflammation supports epileptogenesis.

Another set of *bona fide* miRNAs with links to inflammation is miR-125 and miR-21. miR-125 is generally considered an anti-inflammatory miRNA that targets TRAF6 and inhibits MAPK as well as NF-kB signaling. Furthermore, miR-125 is down-regulated in the hippocampus of PTZ-induced epileptic rat models, while overexpression of miR-125 attenuates seizure activity by suppressing target genes such as, calmodulin-dependent protein kinase IV (CAMK4), and decreases the levels of pro-inflammatory factors *in vivo* [117]. This finding is not surprising given miR-125 regulatees other immune pathways such as the perturbation of B-cell differentiation, and germinal center formation by targeting BLIMP-1 and IRF4. Using a similar rat model, the down-regulation of miR-21-5p in the hippocampus coincided with the up-regulation of STAT3 and onset of epileptic seizures. miR-21 target genes such as caspase-3 and Bax were also highly expressed, while Bcl-2 levels were reduced during this process [113]. This suggests that miR-21 controls onset of epilepsy via the modulation of neuroinflammatory pathways and also via the activation of pro-apoptotic cascades. Interestingly, the use of a miR-21-5p inhibitor *in vivo* resulted in higher IL-6 levels, activation of STAT3, and the apoptotic induction of hyperactive hippocampal neurons. These studies indicate that the proper expression of miR-21 could protect hippocampal neurons from the deteriorating effects of epilepsy.

Overall it is quite clear that miRNAs control the underlying etiology of epilepsy, and continued investigations using animal models will provide sufficient preclinical evidence for the basis of miRNA-based therapies for repairing the epileptic state and restoring brain function. It would be interesting to determine whether the advanced models of epilepsy via the use of gene knockout methodology or site directed mutagenesis will yield genetically linked miRNA pathways that support or abrogate epileptogenesis. Such genetically engineered mouse models of interest include those that contain a missense mutation in *Grin2a*, the genetic ablation of *Kcnq2*, the genetic duplication of *Shank3*, and/or contain a series of missense mutations in *Scn2a*.

### miRNA contribution to ALS

ALS is a fatal neurodegenerative disorder affecting motor neurons in the spinal cord, brainstem, and cerebral cortex [164, 165]. Currently, there are no effective therapeutic options available that delay or slow the onset of ALS. The disease primarily strikes in mid-to-late adulthood, progressively leading to muscle weakness, atrophy, and ultimately respiratory muscle dysfunction, resulting in death [166]. Most ALS cases are sporadic, while only 5%–10% present with a familial history [165]. The median survival is around 3 years, with only 10% surviving beyond 8 years, emphasizing the need for improved diagnostic biomarkers and therapeutic interventions [165]. Repeat expansions in the C9ORF72 gene and mutations in superoxide dismutase 1 (SOD1) are common genetic causes of ALS, while TAR DNA-binding protein (TDP-43) and fused in sarcoma/translated in liposarcoma (FUS/TLS) mutations represent a smaller fraction [165]. Importantly, aberrant miRNA expression profiles are observed in both ALS mouse models and patients, highlighting the potential role of miRNAs in disease progression [165].

Microglia-mediated neuroinflammation plays a complex role in ALS progression. Inflammatory responses are observed in both SOD-1-transgenic mice and ALS patients, influencing disease progression [138]. ALS is gradually being appreciated as an inflammatory disease, since dysfunction of normal microglia and astrocytes contribute to the phenotypes of ALS [138]. For instance, microglia, the immune cells of the CNS, normally promote synaptic maturation and remodeling, control of excessive neuronal activity, and promote oligodendrocyte progenitor cell development. In ALS, the microglia attempt to clear pathological aggregates, at the expense of enhanced proinflammatory cytokine signaling, ROS and nitric oxide production, excessive synaptic pruning, resulting in a neurotoxic state that generates reactive C3^+^ astrocytes. Interestingly, targeting peripheral Ly6Chi monocytes with anti-Ly6C mAb reduces microglial infiltration and modifies the disease course in SOD1 mouse models indicating the monocytes and resident microglia in SOD1 mice influence ALS disease progression [167].

Astrocyte-mediated neuroinflammation also plays a complex role in ALS progression. Astrocytes are instrumental in regulating neuronal synapse release, facilitate neurotransmitter clearance, support glutamate reuptake, and promote antioxidant production. In ALS, these astrocytes are not able to maintain these functions and excessive pro-inflammatory cytokines result in the secretion of neurotoxic factors, increased ROS production, and reduced neurotrophic factors resulting in neuronal degeneration [167]. Many of the phenotypic consequences of astrocyte-mediated neuroinflammation are still being investigated; however, it was recently appreciated that motor neuron cell death is triggered by astrocyte mediated activation of a certain form of programmed cell death called necroptosis. This form of cell death is described as the loss of plasma membrane integrity due to activation of RIPK1 and MLKL. Some investigators are determining if regulators of these kinases could be used as therapeutics to abate the progression of ALS. Here, we argue that miRNAs that target these kinases such as miR-421, miR-381, and miR-425 could serve as effective treatments for ALS.

The greatest supporting evidence for the involvement of miRNAs in ALS etiology is the genetic link to TDP-43. TDP-43 is a member of TAR-binding proteins that are responsible for proper RNA processing. It is known that the generation of a subset of pre-miRNAs requires TDP-43 interactions with the nuclear Drosha complex for the proper cleaving of the cognate parental pri-miRNA. Additionally, cytoplasmic TDP-43 associates with the Dicer complex by binding to the terminal loops of the pre-miRNA facilitating Dicer-mediated cleavage of these pre-miRNAs. Studies by Kawahara et al., clearly show that TDP-43 is indispensable for neuronal outgrowth in cell culture models. Taken together, it is unquestionable that the non-coding RNA regulatory pathway is emerging as a central contributor to ALS pathogenesis.

Mutations in a number of RNA binding genes, such as C9ORF72, SOD1, TDP-43, and FUS result in altered RNA metabolism and correlate with an increased abundance of the protein aggregates similarly found in ALS patients [164]. Specifically, miRNA profiling reveals a series of dysregulated miRNAs in ALS (see Table 1). miR-218 is one miRNA of interest, given the expression of miR-218 is enriched in motor neurons, and was found in the exosomes released from cultured neurons post injury. In cultured primary astrocytes, it was found that miR-218 targets EAAT2, resulting in the modulation of glutamate re-uptake. EAAT2 expression is downregulated in post-mortem brain tissues from ALS patients, and overexpression of EAAT2 in the SOD1 model mentioned above protected animals from L-glutamate induced cytotoxicity. Finally, intracerebroventricular injections of miR-218 inhibitor restored normal astrocyte function as well as GFAP and Cx43 expression. These findings indicate that key miRNAs are vitally important to ALS pathogenesis, and furthermore, that differential expression of a particular miRNA alone is insufficient to predict their involvement with respect to disease progression. Exemplified in the case above, the persistent expression of miR-218 in motor neurons resulted in the chronic excitability of glutamatergic neurons, which was only corrected with the introduction of miR-218 inhibitors.

miR-494-3p is another miRNA that was found to be dysregulated in C9ORF72-ALS derived astrocytes. These astrocytes have a reduction in secreted antioxidant proteins, resulting in a state of oxidative stress and reduced cell survival for co-cultured motor neurons. Restoring the levels of miR-494-3p using miRNA mimics in these C9ORF72-ALS derived astrocytes resulted in healthy co-cultures of motor neurons. The genetic targets and the mechanisms of action of miR-494-3p are still unclear, but some studies point to the regulation of pro-inflammatory mediators such TRAF3, and IKKb/NF-kB resulting in the attenuation of M1 macrophage activation. miR-124 is another miRNA associated with ALS progression, given miR-124 has been linked with motor neuron dysfunction and degeneration in the SOD1 mouse model [168]. Specifically, the overexpression of miR-124 resulted in the early onset apoptosis of wild type motor neurons, while miR-124 inhibitor restored neurite outgrowth mitochondrial dynamics, synaptic signaling, and axonal transport of glutamate in SOD1 motor neurons.

As the field moves towards utilizing miRNAs as biomarkers for ALS onset and progression, some have identified additional mechanistic actions for miRNA-mediated progression of ALS. For instance, the accumulation of glycogen in the lumbar spinal cord of SOD1 ALS mice indicates that certain metabolic abnormalities may be an underlying cause of the neuroinflammatory pathways that support ALS progression [169] (see Figure 2). In support of this notion, GYS1^Nestin-KO^ mice showed significant deficits in learning capacity, as well as altered neurogenesis. Furthermore, normal astrocytes tend to accumulate glycogen similar to muscle cells for utilization during times of high energy demand. Since astrocytes have low levels of glucose-6-phosphate, astrocytes tend to release lactate instead of glucose, which is consumed by the surrounding neurons. Unfortunately, persistent levels of lactate secretion due to overabundant glycogen storage in ALS-astrocytes will also result in the aberrant activation of microglia that in turn secrete pro-inflammatory cytokines TNF-α, IL-6, an IL-1. In support of this hypothesis, miR-338-3p was shown to inhibit glycogenolysis in ALS mice resulting in glycogen accumulation within the spinal cord, while the use of miR-338-3p inhibitors restored some functionality in ALS mice. Interestingly, highly expressed circulating miRNAs such as miR-338-3p, as well as miR-206, miR-183, miR-451, and miR-125a/b are candidate biomarkers for ALS [170–172].

As ALS remains a rare, yet complex disorder with multiple facets of pathogenesis, the potential to cure the disease lies in the continued elucidation of the ncRNA transcriptome which involves the understanding of both miRNA dysregulation and the function of hnRNPs, such as TDP-43. By investigating these pleiotropic regulators in a cell contextual manner, new genetic players that control protein folding, neuroinflammation, glycogen storage, and necroptosis within astrocytes, microglia, and motor neurons will hold the key in determining the next series of therapeutic targets that will slow the progression of ALS. Interestingly, researchers just reported that intravenous application of a subset of anti-inflammatory T-cells attenuates some of the pro-neuroinflammatory phenotypes that hasten ALS progression.

### miRNAs and Parkinson’s disease

Parkinson’s Disease is a neurodegenerative disorder characterized by bradykinesia, rest tremors, and rigidity [173]. While clinical signs will result in a diagnosis of probable Parkinson’s, a definitive diagnosis requires pathological testing to identify the presence of Lewy bodies, which is an abnormal aggregation of α-synuclein protein. The underlying biological mechanisms of Parkinson’s involve the loss of dopaminergic neurons, particularly in the substantia nigra within the basal ganglia. There is no definitive cause of Parkinson’s, but evidence suggests that genetic and environmental factors play a role in its development [174]. One of the contributing factors of Parkinson’s development is microglia cell dysfunction. Microglia are non-neural cells of the CNS that regulate inflammation, regeneration, and metabolism of neurons. Microglia and astrocytes both secrete pro-inflammatory cytokines, when an infection is present, yet the chronic secretion of pro-inflammatory cytokines result in neuroinflammation associated with the development of Parkinson’s [120]. RNA metabolism may also play a role in the pathogenesis of Parkinson’s. Specifically, genes associated with inherited Parkinson’s have been shown to interact with the RNA translation initiation process and RNA translation components [175]. Although there is no cure for Parkinson’s, treatment options focus on providing symptomatic relief, such as drugs that increase dopamine levels in the striatum or operate on dopamine receptors within the brain [176].

Neuroinflammation is believed to contribute to the development of Parkinson’s. In particular, evidence suggests that neuroinflammation perpetuates the death of dopaminergic neurons by activating the microglia and promoting the infiltration of T-cells at sites of neuronal injury [177]. This pro-inflammatory response specific only to dopaminergic neurons implicates a level of cell context specificity analogous to the regulatory functions of miRNA. In fact, dysregulation of microRNA biogenesis appears to disrupt the balance of inflammatory processes in the brain, and results in the heightening of neuroinflammatory cues specifically associated with the Parkinsonian phenotype. The loss of Dicer in striatal dopaminergic neurons using a conditional knockout mouse model resulted in animals having shortened lifespan, an expansion of the resident astrocyte population, yet no neuronal degeneration [178]. However, the loss of Dicer throughout the entire midbrain of these animals resulted in progressive dopaminergic neuronal loss. Furthermore, patients with chromosomal 22q11.2 deletion syndrome, which includes the genetic loss of DGCR8, have a higher occurrence of Parkinson’s as confirmed by pronounced Lewy body formation in post-mortem brain specimens [178]. Additionally, specific miRNAs such as miR-433, miR-16, and miR-7 regulate the genetic pathways associated with α-synuclein abundance, which is a precursor for α-synuclein protein aggregation and Lewy body formation. miR138 and miR-184 have also been reported to control the LRRK2 and E2F1 genes respectively resulting in aberrant lysosomal activity of astrocytes. Understanding the connection between microRNAs and neuroinflammatory processes, such as the generation of reactive astrocytes, may provide valuable insights into the cellular mechanisms of Parkinson’s and lead to the discovery of novel therapeutics to slow disease progression [178] (see Table 1).

#### The involvement of miRNAs in animal models of Parkinson’s

miR-124, a microRNA associated with neurogenesis, has been shown to have decreased expression in animal models of Parkinson’s that have been treated with lipopolysaccharide (LPS), as well as in the 1-methyl-4-phenyl-1,2,3,6-tetrahydropyridine (MPTP) model of Parkinson’s. Yao et al. found that miR-124 targets proteins p62 and p38 to regulate the inflammatory response in the microglia of a Parkinson’s mouse model treated with LPS [179]. When the levels of p62 and p38 were reduced in the microglia, the levels of secreted proinflammatory cytokines were also concomitantly reduced. Furthermore, the decreased levels of p38 promoted autophagy in the surrounding microglia. Autophagy is a normal cellular death response where the surrounding tissue can utilize the recycled cellular material from dying cells. Autophagy is also a normal physiological response to reduce the populations of cells containing α-synuclein protein aggregate formation that is a culprit linked to neuronal degeneration and Parkinson’s disease progression [179]. While autophagy suppresses neuroinflammation, persistent autophagy results in an imbalanced microglial pool resulting in reduced presence of neurotrophic factors and other secreted cytokines that support neurogenesis. Therefore, these results indicate that miRNAs, such miR-124, could be used as therapeutic entities for controlling neuroinflammation, α-synuclein protein aggregation, and abating neuronal degeneration before self destructive cellular processes, such as autophagy are activated in Parkinson’s.

miR-155 is another miRNA found to have increased expression in mouse models of Parkinson’s. Thome et al. found that mice that lacked miR-155 had a reduced proinflammatory response despite adeno-virus-mediated induction of α-synuclein aggregation [180]. Furthermore, the deletion of miR-155 resulted in reduced microgliosis. These findings suggest miR-155 activity plays a key role in the physiological response to α-synuclein aggregation, though it is not clear whether miR-155 prevents this aggregation via promotion of proper protein folding in the ER, regulation of additional chaperone proteins that support aggregate formation, or via the control of an E3 ligase resulting in ubiquitination of α-synuclein. miR-155 is also linked with microglial-mediated inflammation, given TNF-a promotes miR-155 expression, which targets SOCS1 and mitochondrial complex I. The result is aberrant production of cytokines and nitric oxide that triggers neuronal cell death. Therefore targeting miR-155 itself, could be a useful therapeutic strategy to abate the symptoms of Parkinson’s. Interestingly, Caggiu et al. found that the expression of miR-155-5p changed in patients receiving the drug levodopa [181], with patients on the highest dose of levodopa experiencing the greatest down-regulation of miR-155-5p. Because levodopa is a precursor to dopamine, this indicates normal dopaminergic signaling in dopaminergic neurons suppresses pro-neuroinflammatory miRNAs such as miR-155. The addition of a miR-155 antagonist or inhibitors may enhance the efficacy of levodopa by suppressing inflammatory signaling in the microglia and astrocytes in patients with Parkinson’s.

Conversely, miR-375 can be used to protect dopaminergic neurons in Parkinson’s. Cai et al. demonstrated that increased levels of miR-375 in a simulated rat model improved behavioral changes, reduced neuroinflammation and oxidative stress, and decreased dopamine levels [114]. Protection of dopaminergic neurons was achieved through up-regulation of miR-375, specifically by inhibiting the transcription factor specificity protein 1 (SP1), which is involved in neurodegeneration of Parkinson’s. The reverse effect was found when SP1 was up-regulated. Furthermore, Wang et al. used a mouse model to demonstrate that increasing miR-93 expression and reducing protein STAT3 can help protect cells and reduce inflammation in Parkinson’s [115]. Mice exposed to LPS had high levels of STAT3. When expression of miR-93 was increased, STAT3 levels were reduced and inflammation in turn decreased. They found that miR-93 directly targets STAT3, and increasing miR-93 helps to protect cells that produce dopamine in Parkinson’s mice.

#### The mechanistic basis for miRNA-based therapies in Parkinson’s

Given miRNA are pleiotropic regulators of cellular processes, it is not surprising to find a number of gene targets for a specific dysregulated miNA identified in the pathogenesis of Parkinson’s. However, miRNAs are also crucial in regulating a series of genetic cascades that result in a coordinated cellular response, (i.e., go, no go signaling for deciding cell proliferation vs. differentiation). Therefore, the challenge is in understanding which miRNA(s) will have the greatest capacity to support dopaminergic nerve cell survival, a healthy microglial network, while also preventing protein aggregation formation and reduced mitochondrial dysfunction.

Yao et al. demonstrated that miR-124 regulates the expression of mitogen-activated protein kinase kinase 3 (MEKK3) in microglial cells, which regulates the neuroinflammatory response in PD [179]. Knockdown of MEKK3 inhibited microglial activation by regulating the nuclear factor kappaB (NF-κB) signaling pathway. Overexpression of miR-124 decreased the expression of pro-inflammatory cytokines and increased the secretion of neuroprotective factors. One therapeutic possibility is for miR-124 to be delivered via traceable polymeric nanoparticles (NPs). Saraiva et al. found that miR-124 NPs were able to help promote neural stem/progenitor cells and neuroblasts to neurons *in vitro* [182]. The miR-124 NPs also reduced the expression of genes targeted by miR-124. In a mouse model, injecting miR-124 NPs directly into the brain increased the number of neuroblasts that migrated to the olfactory bulb, both in mice with and without PD. The olfactory bulb is typically the first region of the brain to experience accumulation of α-synuclein [182].

miR-7 and miR-153 target the protein α-synuclein. One study investigated the effects of overexpression of these two microRNAs using a PD cell culture model with the neurotoxin MPP+, which causes apoptosis and changes in intracellular signaling pathways. Overexpression of miR-7 and miR-153 was found to protect cortical neurons from MPP+ toxicity by interfering with the MPP+ induced down-regulation of mTOR signaling. Therefore, miR-7 and miR-153 have the protection to protect neurons from apoptosis and may have a critical role in new therapeutic approaches [183]. Another study found that inhibition of the expression of miR-34b and miR-34c increased a-syn levels and aggregate formations [184]. miR-34b and miR-34c had decreased levels in PD patient brains that were in the clinical stage of the disease and were dysregulated in pre-clinical PD brains. The down-regulation of miR-34b and miR-34c results in disturbances in mitochondrial function and increases oxidative stress, leading to disease progression [185].

Taken together, miRNAs have a clear role in regulating the progression of Parkinson’s development. Specifically, miRNA-mediated regulation of α-synuclein aggregate formation, degeneration of dopaminergic neurons, production of reactive oxygen species, promotion of excitotoxicity, and secretion of pro-inflammatory cytokines and microglia activation are all avenues for therapeutic intervention.

### miRNA contribution to Alzheimer’s disease

Alzheimer’s Disease (AD) is the most common neurodegenerative disease (NDD) and the most common form of dementia. The clinical signs of Alzheimer’s include progressive cognitive decline and memory loss, along with difficulties with language processing and problem solving. The cause of Alzheimer’s is unknown, but evidence suggests that a combination of genetics, environment, and lifestyle factors play a role in its development. There is currently no cure for Alzheimer’s, and current therapeutics treat the symptoms of the disease. Alzheimer’s is characterized by the accumulation of amyloid beta plaques and tau protein tangles in the brain, which leads to the loss of synaptic connections and neurons, which in turn leads to neuroinflammation, oxidative stress, and synaptic dysfunction. Specifically, there is increasing evidence that neuroinflammation plays a critical role in the progression of the disease. Increased concentration of proinflammatory cytokines have been found in patients with Alzheimer’s, and PET scans have shown increased microglial inflammation in the brains of patients with Alzheimer’s. Microglia use microRNAs to rapidly respond to inflammation [121].

In the brains of individuals with Alzheimer’s, there are elevated levels of specific microRNAs, including miRNA-9, miRNA-34a, miRNA-125b, miRNA-146a, and miRNA-155. These miRNAs increase in activation when they are around proinflammatory cytokines and amyloid beta, both of which are associated with Alzheimer’s inflammation. These miRNAs are influenced by the protein NFkB, which is elevated in the brains of Alzheimer’s patients. Alexandrov et al. investigated the levels of these miRNAs in the cerebrospinal fluid (CSF) of Alzheimer’s patients as compared to individuals without Alzheimer’s [186]. The results showed that the miRNAs listed above were significantly higher in the CSF of patients with Alzheimer’s than without, indicating their potential role as biomarkers.

#### miRNAs in animal models of Alzheimer’s

miR-155 is a pro-inflammatory microRNA found in the brain. Liu et al. investigated the effects of miR-155 on inflammatory cytokine markers IL-1β, IL-6 and TNF-α in a rat model with Alzheimer’s [187]. They found elevated levels of inflammatory markers and increased miR-155 expression in the hippocampus. When a miR-155 inhibitor was introduced to the rat model, there was a decrease in the expression of the apoptotic protein Caspase-3 in the hippocampus. Apoptotic protein Caspase-3 is an enzyme that is involved in the initiation and execution of apoptosis. The rat model experienced significant cognitive and learning improvement when miR-155 or the inflammatory cytokine receptors were inhibited. This suggests that miR-155 has a crucial role in regulating memory loss through neuroinflammation in Alzheimer’s [187]. Aloi et al. found that deleting miR-155 in the microglia in a mouse model resulted in increased expression of anti-inflammatory genes and therefore reduced plaque and AB buildup in the brain [188]. However, this deletion caused the mice to have hyperexcitability, recurring seizures, and increased seizure related deaths [188]. miR-155 has therapeutic possibilities for Alzheimer’s. Zheng et al. [189] studied the effects of the intravenous drug Propofol on microglial activation (M1 activation) using a mouse model. They found that propofol suppressed the expression of miR-155, suggesting that propofol inhibits neuroinflammation by reducing levels of miR-155.

Song et al. [190] found that miR-30b is a microRNA that is highly up-regulated in the brains of patients with Alzheimer’s and mouse models. Increasing expression of miR-30b in the hippocampus region of wild-type mice impairs cognitive functions, similar to mice with AD. Conversely, reducing levels of miR-30b in mice with simulated Alzheimer’s prevents cognitive decline. They also found that miR-30b is up-regulated by proinflammatory cytokines and AB42. These findings suggest that miR-30b that is dysregulated leads to cognitive and synaptic decline, indicating that reversal of this dysregulation could have therapeutic advantages.

miR-124 is thought to directly affect the protein PTPN1, which is essential for normal brain function. Elevated levels of miR-124 were found in the hippocampus of a mouse model. When miR-124 was increased or PTPN1 was decreased in the mouse model, the mice developed changes similar to those with AD. When the balance of miR-124 and PTPN1 was restored, these changes improved. This shows that the miR-124/PTP1B pathway has a role in brain dysfunction in the development of AD, and reconstructing this pathway could improve cognitive functions in patients with AD [191].

#### Mechanisms of action for the miRNA mediated Alzheimer’s phenotype

Not surprisingly, there are a number of clinical criteria associated with dementia that are distinct from Alzheimer’s. Understanding the clinical description of the symptoms associated with these various dementias, will better enable molecular biologists to match the underlying genetic pathways that could be the instigator of these dementias. In one example, frontotemporal dementia (FTD) is similar to Alzheimer’s yet does not present with patient hallucinations nor loss of spatial orientation. microRNAs also have the potential to help discriminate AD from other types of dementia, such as frontotemporal dementia (FTD), more commonly known as Pick’s disease. FTD is a rare neurodegenerative disorder that primarily affects the frontal and temporal lobes of the brain, and is characterized by the progressive deterioration of behavior, language, and personality. FTD has similar clinical symptoms to other dementias such as Alzheimer’s, making diagnosis difficult and misdiagnosis common [192]. FTD is associated with accumulation of proteins such as tau and TDP-43 in the brain. Specifically, Martinez et al. [193] focused on how miRNA biomarkers found in CSF and blood serums helped distinguish frontotemporal dementia (FTD) from Alzheimer’s and ALS. They found miR-223-3p, miR-15a-5p, miR-22-3p, and miR-124 to all be potential biomarkers for FTD in comparison to Alzheimer’s, ALS, and controls.

Vascular cognitive impairment dementia (VCID) is caused by inadequate cerebral blood flow that results in brain damage. Unlike Alzheimer’s, VCID is both preventable and treatable [194]. microRNAs play an important role in the dysfunctional mechanisms of VCID, including blood-brain barrier dysfunction, apoptosis, oxidative stress, and neuroinflammation. A multiple microinfarction (MMI) model was used to simulate vascular dementia in mice to investigate the role of miR-126, which regulates vascular function. Mice with reduced endothelial miR-126 experienced cognitive impairment, decreased blood flow, inflammation, and glymphatic dysfunction, showing miR-126's role in cognitive deterioration [116].

With respect to Alzheimer’s, miR-132 is known to regulate neuron plasticity and has been shown to be reduced in brains affected with Alzheimer’s. When this gene is deleted in a mouse model, Alzheimer’s deposition is increased. A possible protein that miR-132 might regulate is C1q, which has increased expression in the synapses of Alzheimer’s individuals. When mice were exposed to either miR-132 agonists and/or C1q inhibitors, synaptic proteins increased significantly [195]. Moreover, intranasal delivery of miR-132 in a dementia mouse model could partially improve the cognitive functions of mice, and promote the significant increase of synaptic proteins PSD95 and Synapsin-1.

Conversely, miR-125b may increase neuroinflammation resulting in Alzheimer’s pathogenesis. miR-125b is found to be elevated in the cerebrospinal fluid of Alzheimer’s patients. miR-125 reduces the level of Bcl-W, which prevents apoptosis. It also directly targets DUSP6 and PPP1CA, which are phosphatases. When these phosphatases are decreased using miR-125b, tau hyperphosphorylation increases. In a mouse model, this was found to hinder learning and cognition [111]. Zhao et al. [196] additionally found increased levels of miRNA-34a in patients with Alzheimer’s which affect the expression of the TREM2 receptor. Reduced expression of TREM2 impairs the ability of microglia to perform phagocytosis and leads to increased production of proinflammatory cytokines. However, when an anti-miR-34a molecule was used in animal models, it prevented this harmful effect and TREM2 expression was restored, suggesting a potential therapeutic target.

Together, these findings shed light on the involvement of specific miRNAs in the pathogenesis of Alzheimer’s and their impact on various inflammatory processes and immune responses. Further research in this field could potentially lead to the development of targeted therapies aimed at modulating these miRNAs and mitigating the neuroinflammatory component of Alzheimer’s.

### miRNAs and Huntington’s disease

Huntington’s disease is a rare neurodegenerative disorder caused by an autosomal dominantly-inherited CAG-trinucleotide repeat-expansion encoding for glutamine in the huntingtin (*HTT*) gene, leading to protein misfolding and aggregation of aberrant proteins [153, 155, 197]. It is characterized by cognitive, motor, and psychiatric disturbance. At the cellular level, mutant HTT results in neuronal dysfunction and death through a number of mechanisms, including disruption of proteostasis, transcription and mitochondrial function, and direct toxicity of the mutant protein [197].

Transcriptional dysregulation is one of the major pathogenic processes in HD. Transcriptional alterations are present in Huntington’s mice before the onset of disease symptoms and in asymptomatic HD-mutation humans, suggesting that perturbed gene expression is one of the causes of pathogenesis. Zsindely et al. [153] investigated miRNAs that are misregulated in a *Drosophila* model of Huntington’s. miRNAs function as post-transcriptional regulators of gene expression. Therefore, miRNA dysregulation introduces a second layer of altered gene regulatory mechanisms besides the transcriptional misregulation of mRNAs in HD and can pinpoint those molecular processes that are part of the response to mHtt-induced proteopathic stress [153].

They determined the functional effects of three down-regulated (dme-mir-137, dme-mir-219, and dme-mir-1010) and two up-regulated (dme-mir-10 and dme-mir-305) miRNAs on mHtt-induced pathology. Overexpression of both dme-mir-137 and dme-mir-1010 ameliorated mHtt-induced phenotypes, indicating that the down-regulation of these miRNAs is pathological. Overexpression of dme-mir-219, on the other hand, was detrimental both in the Huntington’s model and in control flies, suggesting that in this case, the observed phenotypic outcome is due to a toxic effect of dme-mir-219 overexpression that is independent of Huntington’s pathology. Overexpression of dme-miR-10 also exacerbated all analyzed phenotypes of Huntington’s flies, and it also had a mildly negative effect on the eclosion rate of control flies. This suggests that flies are sensitive to the level of dme-mir-10-5p, and increased expression of this miRNA might be a part of the molecular basis of pathology in the HD model. Overexpression of dme-mir-305 alleviated all analyzed phenotypes of Huntington’s flies and had significant detrimental effects on the viability, longevity, and motor activity of control flies [153].

Fukuoka et al. [154] investigated the use of miR-132 as a potential treatment for Huntington’s disease (HD). A recombinant adeno-associated virus (rAAV) miRNA expression system was used to deliver miR-132 to the R6/2 mice. Findings demonstrated that giving Huntington’s mice access to miR-132 enhanced motor function and lengthened their lives. The therapy had no impact on the expression of disease-causing mutant HTT genes and their byproducts. Supplementing with miR-132 may be a treatment strategy for easing Huntington’s symptoms and delaying the onset of the disease without specifically addressing the underlying genetic etiology. Although it might not offer a permanent cure for Huntington’s, it exhibits promise as a prospective therapeutic approach [154].

Degeneration of the medium spiny neurons (MSNs) in the striatum of the basal ganglia is the key pathological feature of Huntington’s [198]. Using a high-throughput miRNA interaction reporter assay (HiTmIR) to study the interactions between miR-34a-5p and HD associated genes, Hart et al. (2023) identified NDUFA9, TAF4B, NRF1, POLR2J2, DNALI1, HIP1, TGM2, and POLR2G as direct miR-34a-5p target genes. Down-regulation of miR-34a-5p in MSNs of R6/2 mice and human HD brain samples might contribute to an elevated intracellular Ca^2+^ level, increased UPR and ROS levels resulting in a pronounced activation of apoptosis. These results lay the ground for future therapeutic interventions using miRNA-34 [155].

Although Huntington’s mainly affects the CNS, Diez-Planelles et al. [199] hypothesized that ubiquitous expression of mutant HTT contributes to disturbances in circulating miRNA profile in symptomatic Huntington’s patients, reflecting changes in both the central nervous system (CNS) and peripheral tissues. To test their hypothesis, they analyzed plasma samples from symptomatic patients with 40–45 CAG repeats in the *HTT* gene, the most common range among Huntington’s patients, screening for changes in the Huntington’s circulating miRNA profile of healthy matched controls. The Unified Huntington’s Disease Rating Scale and Total Functional Capacity, two disease severity indicators, were found to be linked with the expression levels of specific circulating miRNAs. Additionally, the study found that Huntington’s patients had an overexpression of circulating miRNAs that regulate metabolism. According to these results, circulating miRNA profiles may be used as possible biomarkers for tracking the development of the disease and developing customized treatments for Huntington’s patients [199].

The expression of miRNAs in the prefrontal cortex (BA9) of Huntington’s patients and normal subjects was examined by Hoss et al. [156] Next-generation miRNA sequence analysis was performed on tissue samples from 26 Huntington’s, 2 Huntington’s gene-positive, and 36 control brains. Neuropathological information available for all Huntington’s brains included age at disease onset, CAG-repeat size, Vonsattel grade, and Hadzi-Vonsattel striatal and cortical scores, a continuous measure of the extent of neurodegeneration. Linear models were performed to examine the relationship of miRNA expression to these clinical features, and messenger RNA targets of associated miRNAs were tested for gene ontology term enrichment.

75 differentially-expressed miRNA’s were identified in the tissue samples. Nine were significantly associated with Vonsattel grade of neuropathological involvement and three of these, miR-10b-5p, miR-10b-3p, and miR-302a-3p, significantly related to the Hadzi-Vonsattel striatal score (a continuous measure of striatal involvement) after adjustment for CAG length. Five miRNAs (miR-10b-5p, miR-196a-5p, miR-196b-5p, miR-10b-3p, and miR-106a-5p) were identified as having a significant relationship to CAG length-adjusted age of onset including miR-10b-5p, the mostly strongly overexpressed miRNA in Huntington’s cases. These results provide insight into striatal involvement and support a role for these miRNAs, particularly miR-10b-5p, in HD pathogenicity. The miRNAs identified in our studies of postmortem brain tissue may be detectable in peripheral fluids and thus warrant consideration as accessible biomarkers for disease stage, rate of progression, and other important clinical characteristics of Huntington’s [156].

Chang et al. [157] examined 13 miRNA’s (miR-1, mirR-9, miR-9*, miR-10b, miR-29a, miR-29b, miR-124a, miR-132, miR-155, miR-196a, miR-196b, miR-330, and miR-615) that previously demonstrated alterations in the brains of Huntington’s patients or that are potential regulators of differentially-expressed genes in brains of Huntington’s patients, to examine their expression in the peripheral leukocytes of Huntington’s patients healthy controls. Expression levels of miR-9* were much lower in Huntington’s patients compared with those in healthy controls, suggesting that miR-9* may be involved in the pathogenesis of Huntington’s. miR-9 is highly conserved across vertebrate species and shows brain-specific expression. The complementary strand miR-9* is also crucial for brain development. These findings suggest that miR-9*-mediated gene regulation is important for synaptic plasticity and memory, both of which play an important role in the generation of abnormal movements and psychiatric abnormalities in Huntington’s [157].

## Conclusion

This article examines a number of disorders connected to miRNA levels and how they control neuroinflammation. In the future, therapeutics that target particular miRNAs might possibly be created to treat neurological problems. Some miRNA have already been suggested as potential biomarkers. By targeting miRNAs that are involved in neuroinflammation, the severity of many neurological disorders might be reduced [200]. As an example, miRNAs that promote neurodegeneration [201], have been demonstrated to activate TLR7 in neurons and cause neurotoxicity [201]. According to Lehmann et al., the increase in pro-neurodegenerative miRNAs may be an indicator of aberrant transcriptome signaling, or improper chromatin remodeling that alters the entire epigenetic landscape of normal microglial cells. This notion makes some intuitive sense, given similar miRNAs are found to be dysregulated in a wide variety of neurological disorders with vastly varied clinical manifestations. Therefore, while the immediate goal is to implement miRNA to slow the progression of neurodegenerative illnesses, researchers should be assessing the entirety of the epigenome so as to elucidate the neuroinflammatory cues that promote disease specific phenotypes.

From the diagnostic perspective, researchers are able to distinguish between various forms of neurodegenerative illness using serum miRNA levels [202]. Researchers examined a collection of miRNAs in a particular patient cohort that were linked to Alzheimer’s disease, Parkinson’s disease, vascular dementia, and mixed vascular-parkinsonism [202]. The analysis discovered dysregulated miRNAs in comparisons of various diseases. The findings indicate that dysregulation of miR-34b, miR-125b, and miR-130b is related to Alzheimer’s disease and that altered expression of miRNAs such miR-15b, miR-24, miR-142-3p, miR-181c, and miR-222 is connected with Parkinson’s disease [202]. When these miRNAs were combined into an indicator, as opposed to when they were used individually, the diagnostic accuracy was enhanced, according to multivariable ROC curves [202]. Circulating miRNAs that are linked to proteins or exosomes are stable, which makes them desirable as diagnostic biomarkers [202].

So how will a miRNA be implemented? An individual miRNA can have multiple targets, regulating several genes in a single pathway or single genes in several pathways. For example, genetic deletion of brain-expressed miR-128 in mice resulted in up-regulation of more than a thousand mRNA transcripts, of which 154 were predicted targets of miR-128. This multi-targeting property of miRNAs has advantages for disease modification in neurological diseases because it offers the possibility to disrupt several pathological processes at once. However, it also increases the potential for unwanted or unanticipated side-effects of any miRNA-based therapy [160]. Unfortunately the hurdle we must overcome in the therapeutics arena is the acceptance that multigenic disorders such as Alzheimer’s or epilepsy are going to require pleiotropic therapeutics that can function synergistically to correct or reverse the underlying faulty genetic pathways of the disease.

The specific hurdle to neurocentric miRNA drug delivery is the blood brain barrier, which prevents miRNAs from entering the CNS and reduces their efficacy [203]. Roy et al. [203] suggested two methods to improve the delivery of miRNAs and get around this obstacle. The first includes utilizing miRNA mimics (agonists) that are packaged into lipid nanoparticles so as to facilitate uptake of the miRNA into the CNS. In the second method, miRNA delivery could be implemented via exosomes carrying surface targeting molecules to facilitate cell specific uptake of the miRNAs therapeutic [204]. This strategy could also be used to aid in the transport of the therapeutic through the blood brain barrier. Additional research is still required to determine how these approaches might be implemented to treat the neurological conditions mentioned in this review.
